# Internet treatment of sexually transmitted infections – a public health hazard?

**DOI:** 10.1186/1471-2458-7-333

**Published:** 2007-11-15

**Authors:** Roberto Vivancos, Silke Schelenz, Yoon K Loke

**Affiliations:** 1 School of Medicine, Health Policy and Practice, University of East Anglia, Norwich NR4 7TJ, UK

## Abstract

**Background:**

Owing to the stigma associated with sexually transmitted infections, patients may prefer to keep their illness private, and choose instead to try self-treatment remedies from the internet. However, such remedies may prove hazardous if the sellers do not provide detailed advice on adverse effects, or on avoiding transmission and re-infection. We conducted an internet search to determine the availability of treatments for STIs and the nature of information provided by vendors of these treatments.

**Methods:**

We conducted a systematic internet search using five different search engines in February 2007. The search term included the words "self treatment" and the name of six different common STIs. We visited the vendors' websites and recorded any information on the formulation, adverse effects, cautions, and prevention of infection.

**Results:**

We identified a total of 77 treatments from 52 different companies, most of which were sold from the UK and US. The available remedies were predominantly for topical use and consisted mainly of homeopathic remedies. Only a small proportion of the web-listed products gave details on adverse effects, contraindications and interactions (22%, 25% and 9% respectively). Similarly, web vendors seldom provided advice on treatment of sexual contacts (20% of chlamydia and 25% of gonorrhea treatments) or on preventive measures (13%). Conversely, evidence of effectiveness was claimed for approximately 50% of the products.

**Conclusion:**

While treatments for certain STIs are widely available on the internet, purchasers of such products may potentially suffer harm because of the lack of information on adverse effects, interactions and contra-indications. Moreover, we consider the paucity of preventive health advice to be a serious omission, thereby leading to patients being needlessly exposed to, and potentially re-infected with the causative pathogens.

## Background

Although the incidence of sexually transmitted disease (STIs) has decreased over the past few decades in Western Europe, recent years have seen a reversal of this trend [1]. For instance, there has been an increase in notifications of *Chlamydia trachomatis*, secondary syphilis, genital warts and genital herpes in the UK over the past years [2].

One of the major barriers in the management of sexually transmitted infections is the associated stigma that may prevent patients from seeking professional medical care [3-5]. While media campaigns may lead to enhanced general awareness of STIs, patients who shun the spotlight may turn to lay systems of care in search of a quick and easy cure. Here, the internet can offer a sense of privacy, making it a useful, confidential resource for information in stigmatized conditions, and reaching subgroups that may not normally be captured through other means [6]. Recent research has highlighted the widespread Internet availability of self test kits for a number of conditions, including Chlamydia [7]. The potentially easy access to diagnostic tests and treatments from the privacy of one's home may make the internet an appealing avenue for patients seeking remedies for their STIs.

One of the major concerns regarding internet purchases is whether the appropriate safeguards have been put in place to protect the patient. Treatment of STIs is not a simple one-off solution – the remedies will inevitably fail if users are not advised on minimizing re-infection and concurrent treatment of sexual contacts. Moreover, licensed drugs are tightly regulated and come with government approved information leaflets that help patients to check that the medication is indeed safe and suitable for them. This may not be the case for Internet purchases.

As such, we surveyed the availability of treatments for common STIs on the Internet, and evaluated the product information and public health advice given by the vendors of such treatments.

## Methods

We conducted a systematic search of the internet using ' [infection] self treatment' as the search term, where [infection] was substituted by one of the following STIs: genital warts, HPV, genital herpes, chlamydia, gonorrhea and syphilis. There are a variety of search engines available for the retrieval of information from the internet. To increase the likelihood of finding treatments for STIs, we used five search engines: *Google*™, *Ask*™, *Yahoo!*^®^, *MSN*^® ^and *Clusty*. The most popular of this is Google, which is reported to cover 75% of the internet; the first four together power 95% of the internet searches in the UK [8]. *Clusty *was added for completeness. The search was conducted in February 2007.

Eighty percent of people searching the internet would only use the first two pages retrieved by the search [9]. In order to replicate this typical behavior, we opened all the links on the first two pages retrieved by the search engine, plus all the sponsored links on those pages. For *Clusty*, we also explored the first two pages of all the relevant 'clusters'. We visited websites that offered treatment of STIs (with or without offering concurrent diagnostic tests) and retrieved product data onto a database with predefined fields (Table 1). We collected information on the type of treatments, including its compounds if reported, the dose and length of treatment, the cost, whether interactions, contraindications or side effects were reported, and if advice regarding prevention of transmission or treatment of sexual partners was offered.

**Table 1:** Data collected for each product.

• Product name • Manufacturer (or company selling the product) • Country where sold from • Active component • Formulation • Price per item (smallest pack size) • Side effects • Contraindications • Interactions • Medical consultation/advice offered • Partner treatment recommended • Prevention advice

The key criteria for inclusion onto the database were that the websites were selling products for the treatment of STIs; we also included website that sold information (rather than physical remedies) on how to treat the conditions (i.e. e-books). We only researched the websites and the products offered up to the point where payment was required. On completion of the database, we aggregated the collected data and descriptively summarized the general characteristics of the available treatments.

## Results

A total of 77 products from a total of 52 companies were identified in the search (Table 2). These products were shipped from six different countries. The majority (61%) were from companies based in the United States, followed by the United Kingdom (17%). The cost of the smallest pack for each of the treatments ranged from $14.95 to $290 (mean $51.76). The average cost did not differed significantly between the different STIs.

**Table 2:** details of the products identified by the conditions treated (search conducted in February 2007).

	*Chlamydia*	*Genital herpes*	*Genital warts*	*Gonorrhoea*
Number of products	5	39	29	4
Treatment recommended by UK treatment guidelines [11–15]	3†	0	4	3†
Number of companies	4	20‡	25‡	3
Country (shipping from)				
China	-	1 (3%)	1 (3%)	-
EU (unspecified)	-	2 (5%)	1 (3%)	-
India	-	1 (3%)	-	-
New Zealand	1 (20%)	1 (3%)	1 (3%)	-
UK	3 (60%)	3 (8%)	4 (14%)	3 (75%)
US	1 (20%)	29 (74%)	16 (55%)	1 (25%)
Not specified	-	2 (5%)	6 (21%)	-
Average (range) package cost§	58.28 (19.95–87.89)	44.84 (14.95–290)	56.99 (15.95–229)	73.22 (39–107.42)
Side effects reported*	2 (40%)	6 (15%)	7 (24%)	2 (50%)
Contraindications reported*	2 (40%)	6 (15%)	9 (31%)	2 (50%)
Interactions reported*	2 (40)%	2 (5%)	1 (3%)	2 (50%)
Consultation offered*	3 (60%)	13 (33%)	6 (21%)	3 (75%)
Recommended medical advice*	3 (60%)	7 (18%)	5 (17%)	3 (75%)
Partner treatment*	1 (20%)	-	2 (7%)	1 (25%)
Prevention advice*	2 (40%)	2 (5%)	4 (14%)	2 (50%)
Claims of effectiveness*	3 (60%)	24 (62%)	9 (31%)	2 (50%)

* Value is number and percentage of products; ‡ each product sold on e-bay is counted as belonging to a separate company; §costs are given in USD (USD:GBP = 0.512/USD:EURO = 0.763); † treatment consists of an unspecified antibiotic

The types of active compounds present in the products identified were: homeopathic (73%), mainly for genital warts and herpes; antibiotics (8%) for the treatment of Chlamydia and Gonorrhea; and some chemical compounds (5%), like podophyllum and imiquimod for the treatment of genital warts. Information on the active compound was not available for 14%. Of the homeopathic remedies two (for the treatment of herpes) contained dilutions of viruses (including a variety of herpes viruses, cytomegalovirus, Epstein Barr virus and poliomyelitis) that claimed to stimulate natural immunity to clear the infection. Two other websites offered e-books on proven treatments of genital warts with remedies that could be made with products home available; but there was not enough information on the websites to determine the exact nature of these products.

Overall, side effects were discussed in 22% of the products, contraindications in 25% and interactions with other drugs in 9%. The reporting of these was better for treatments of bacterial infections for which it was also more likely that an online medical consultation was offered or that advice to seek medical attention in case of treatment failure would be given if treatment failed. Where side effects were discussed this related to skin irritation; also the commonest contraindication, when mentioned, was pregnancy and breastfeeding. However, in the seven products where drug interactions were available four were related to antibiotics in which a pre-treatment form or some sort of virtual consultation was required.

Inversely, there were claims of beneficial properties in almost 50% of the products, either by stating its effectiveness or by referring to approval by a regulatory body or the compliance with manufacturing regulations. Where studies were cited, these were hard to track down or referred to empirical research. Testimonial from other patients was widely used by most of the websites as a form of qualitative evidence.

Recommendation that sexual partners should also be treated or advice regarding prevention (e.g. use of condoms, abstinence while on treatment, testing for other STI, etc.) was rarely given (13% had preventive messages). Partner treatment was recommended in 20% of treatments for chlamydia and 25% for gonorrhea (Table 2).

## Discussion

The internet may provide a sense of privacy, as highlighted by behavioral research into stigmatized issues [6]. As a result people may turn to the internet to seek help for a STI. In our study we identified many products on offer for symptomatic conditions with topical manifestations, such as genital warts and herpes. Conversely, there were far fewer treatments available for conditions such as Chlamydia or Gonorrhea infection that may not be obvious to the person suffering them. This may be because these conditions require specific testing to identify the causal agent or because a larger proportion of patients may be asymptomatic [10].

The cost of treatments on offer ranged from $14.95 to $290, making some of the treatments relatively affordable in comparison with the current cost of a prescription or the actual cost of some of the treatments recommended by accepted treatment guidelines [11-15]. On the other hand, some treatments were relatively expensive, which suggest that treatment cost may not be an issue for some.

Active components of the different treatment varied considerably. These were mainly homeopathic or herbal remedies. Although there is some empirical evidence that compounds like lemon balm and lysine may have antiviral properties [16,17], there is not enough information in the websites that we identified to judge whether treatments are effective or not. Some vendors provided examples of clinical studies, but the cited evidence was limited to in-vitro research in the best cases, or poor quality or doubtful studies in others. None of the websites offered information about well conducted randomized control trials in humans. While we do have serious misgivings around the lack of evidence on efficacy, we are also equally concerned at the lost opportunity for essential public health advice on prevention and control of transmission, the absence of which will negate the expected benefits on any treatment.

There is evidence that co-infection with more than one STI can occur [18]. Generally we found that no advice was given to test for concomitant infections. Also worryingly, most of the vendors' websites we identified did not display information on prevention of transmission, such as use of condoms or abstinence while on treatment. Nor did they recommend notification, testing and treatment of sexual partners. The glaring lack of advice on avoiding re-infection, and treatment of contacts means that some of these remedies are doomed to fail. Indeed, this may contribute to greater individual hazard, and risk to the public from greater spread of the STIs.

The majority of the products were from companies based in the US or the UK, where there is good regulation of drugs and therapeutics. However, as some of the products sold through the internet could be classed as herbal extracts, topical applications or even beauty products, they may be overlooked by regulations requiring a prescription from a qualified health professional. The lack of information on side effects, contraindications or interaction is in contrast with the many unsubstantiated claims of effectiveness; this may mislead potential buyers to believe these treatments are better than recommended pharmacological treatments. Moreover, the absence of trial data on these products means that patients may inadvertently be paying for, and using products which are unsafe or unsuitable for them.

This study is limited by the lack of information on how many people access and buy products to self treat STIs via the internet. We are only able to comment on the amount of products and their affordability and hence speculate that a market exists for these products. Anecdotally, during the time that we were conducting the search a total of 21 items of four different products we identified for genital warts or genital herpes were sold through eBay; whereas the number of people that had viewed each product on sale ranged between 61 and 263 (based on values on the eBay counter for each page). Of the 4 products sold through a single eBay trader, there were at least 29 confirmed buyers over the past 90 days who left positive feedback. This is likely to be a significant underestimate though as purchasers of such products may prefer not leave their details on the seller's feedback page.

Also, this study only focuses on one source of alternative treatment for STIs (i.e. the internet) and it does not address questions regarding accessibility and usage of these alternative sources. While we did not actually purchase any products, other researchers have reported success in buying both prescription drugs and alternative therapies from Internet merchants[19].

Unfortunately, we are unable to explain the characteristics of people that may access treatment for STIs in this way, but merely speculate that if treatments are being sold and bought on the internet means that there are people willing to use alternative ways of accessing treatment. It is possible that people who use the internet for such therapies are already well-versed with preventative measures and may be purchasing the treatment for both themselves and their partners. Conversely, the lack of information from web traders on prevention may well be a cynical way of ensuring repeat visits from their customer base. Further research may be needed to determine the role that alternative therapies and lay systems play in the treatment of STIs and therefore be able to asses the overall impact that these may have in their epidemiology and transmission control.

Regulatory agencies' websites in the UK and US warn consumers of the problems of purchasing treatments through the internet and of the doubtful quality of some of the products [20,21]. It has also been suggested that doctors need to advise their patients against using drugs bought on the internet [22]. We argue that some patients with STIs may not even present to a doctor at all. Indeed, government information campaigns that increase the awareness of STIs may potentially increase the market for STI remedies on the Internet, given the stigmatized nature of STIs and the relative convenience of online purchases.

The marketing and professional regulations of some of these products or companies may not be as tight as those provided by registered pharmaceutical companies and the medical establishment, thus resulting in lack of information about side-effects, contraindications or interactions. Furthermore, because of the lack of contact with health care professionals, a valuable opportunity for patient education and prevention advice is lost.

## Conclusion

As regulation of vendors on the Internet is unlikely to ever be successful, we propose that the public should be educated on the dangers of using self-treatment of STIs, particularly where preventative measures are essential in stopping re-infection. Government agencies could explore the possibility of using the same search engines that are used by potential customers of these products, ensuring that similar searches also return prominent links to promotional sites warning of the dangers and giving appropriate preventive advice.

## List of abbreviations

HPV Human Papilloma Virus

STI Sexually transmitted infection

UK United Kingdom

US United States

## Competing interests

The author(s) declare that they have no competing interests.

## Authors' contributions

All authors participated in the design and data collection. RV drafted the manuscript with input from SS and YKL. All authors have read and approved the final manuscript

## Pre-publication history

The pre-publication history for this paper can be accessed here:


